# Manipulating the reliability of target-color information modulates value-driven attentional capture

**DOI:** 10.3758/s13414-024-02878-7

**Published:** 2024-03-27

**Authors:** Nicole B. Massa, Nick Crotty, Ifat Levy, Michael A. Grubb

**Affiliations:** 1 Trinity College, Hartford, CT USA; 2 Mass General Brigham, Boston, MA USA; 3 Yale University, New Haven, CT USA

**Keywords:** Attention, Attentional capture, Attention in learning

## Abstract

Previously rewarded stimuli slow response times (RTs) during visual search, despite being physically non-salient and no longer task-relevant or rewarding. Such value-driven attentional capture (VDAC) has been measured in a training-test paradigm. In the training phase, the search target is rendered in one of two colors (one predicting high reward and the other low reward). In this study, we modified this traditional training phase to include pre-cues that signaled reliable or unreliable information about the trial-to-trial color of the training phase search target. Reliable pre-cues indicated the upcoming target color with certainty, whereas unreliable pre-cues indicated the target was equally likely to be one of two distinct colors. Thus reliable and unreliable pre-cues provided certain and uncertain information, respectively, about the magnitude of the upcoming reward. We then tested for VDAC in a traditional test phase. We found that unreliably pre-cued distractors slowed RTs and drew more initial eye movements during search for the test-phase target, relative to reliably pre-cued distractors, thus providing novel evidence for an influence of information reliability on attentional capture. That said, our experimental manipulation also eliminated *value-dependency* (i.e.*,* slowed RTs when a high-reward-predicting distractor was present relative to a low-reward-predicting distractor) for both kinds of distractors. Taken together, these results suggest that target-color uncertainty, rather than reward magnitude, played a critical role in modulating the allocation of value-driven attention in this study.

**Supplementary Information:**

The online version contains supplementary material available at 10.3758/s13414-024-02878-7.

## Introduction

From kaleidoscopic sensory input to a parade of internal thoughts, each moment is replete with stimuli competing for attention. While much is known about attention and its impact on visual perception (Carrasco, 2011), recent data have challenged a long-standing dichotomy in the field. Generally, researchers conceptualize attentional control as being either top-down (allocated in a goal-directed manner) or bottom-up (elicited reflexively by salient environmental stimuli). But non-salient stimuli can reflexively draw attention and actively interfere with top-down goals, generating selection biases that are neither top-down nor fully bottom-up. Instead, such biases rely on one’s unique history of selection (Anderson et al., 2021; Awh et al., 2012; Failing & Theeuwes, 2018).

In the paradigmatic example that spawned an entirely new subfield of attention research, a distractor rendered in a color that previously signaled monetary reward slows responses and captures eye movements during visual search (Anderson et al., 2011; Anderson & Yantis, 2012). Such value-driven attentional capture (VDAC) occurs despite the distractor being physically non-salient, task-irrelevant, and no longer rewarding.

### Value-driven attentional capture (VDAC) as the paradigmatic example of experience-driven attention

In their seminal publication (Anderson et al., 2011), Anderson and colleagues presented a method for generating and measuring VDAC during visual search. In the VDAC paradigm, observers complete two phases: a training phase and a test phase. During the training phase, observers search for a color-defined target (a red or green circle) and receive a high or low monetary reward for correctly discriminating the orientation of a line inside the target (two-alternative forced-choice: vertical or horizontal). Because the color-reward pairings are consistent, observers learn, over many training trials, to associate high or low reward with each target-defining color. In the test phase, observers search for a shape-defined target (e.g., diamond among circles) and again discriminate the orientation of the line contained inside. Colors of individual elements are irrelevant, and responses are no longer rewarded. But on half the trials, one of the distractor elements is rendered in a color that previously signaled high or low monetary reward. Search is conducted under time pressure in both phases, and VDAC is typically quantified by a change in mean response time (RT) during the test phase: orientation judgments of the line inside the unique shape are slowed when a high-reward distractor is present, relative to when a low-reward distractor or no reward distractor is present.

Ample evidence now points to dopaminergic reward prediction errors as playing a crucial role in the generation of VDAC (Anderson et al., 2014, 2016; Sali et al., 2014). Seminal research on dopamine-mediated reward learning (e.g., Schultz et al., 1997) has demonstrated that unexpected rewards generate a response from midbrain dopaminergic neurons. When a stimulus in the environment precedes and reliably predicts the delivery of reward, the same dopaminergic neurons begin to respond to the presentation of this reward-predicting stimulus. If the delivered reward is different than the expected reward, a reward-prediction error response is generated, which can be used to update future predictions, and thereby facilitate learning (for a thorough treatment of reward-prediction based learning, see: Daw & Tobler, 2013; Schultz, 2015). Thus, in the context of the VDAC training phase, the color of the search target serves as a reliable predictor of either high or low impending reward, and in turn, elicits a dopamine-mediated reward prediction. In the test phase, magnitude-dependent reward-predictions are theorized to still be elicited when a distractor is rendered in a former target color, drawing attention to task-irrelevant, physically non-salient elements of the visual array and slowing search for the test-phase target (for a detailed discussion of reward-prediction in the VDAC context see: Anderson, 2018; Anderson et al., 2021).

### Modulating VDAC with higher-order conditioning – rationale

**Fig. 1:**
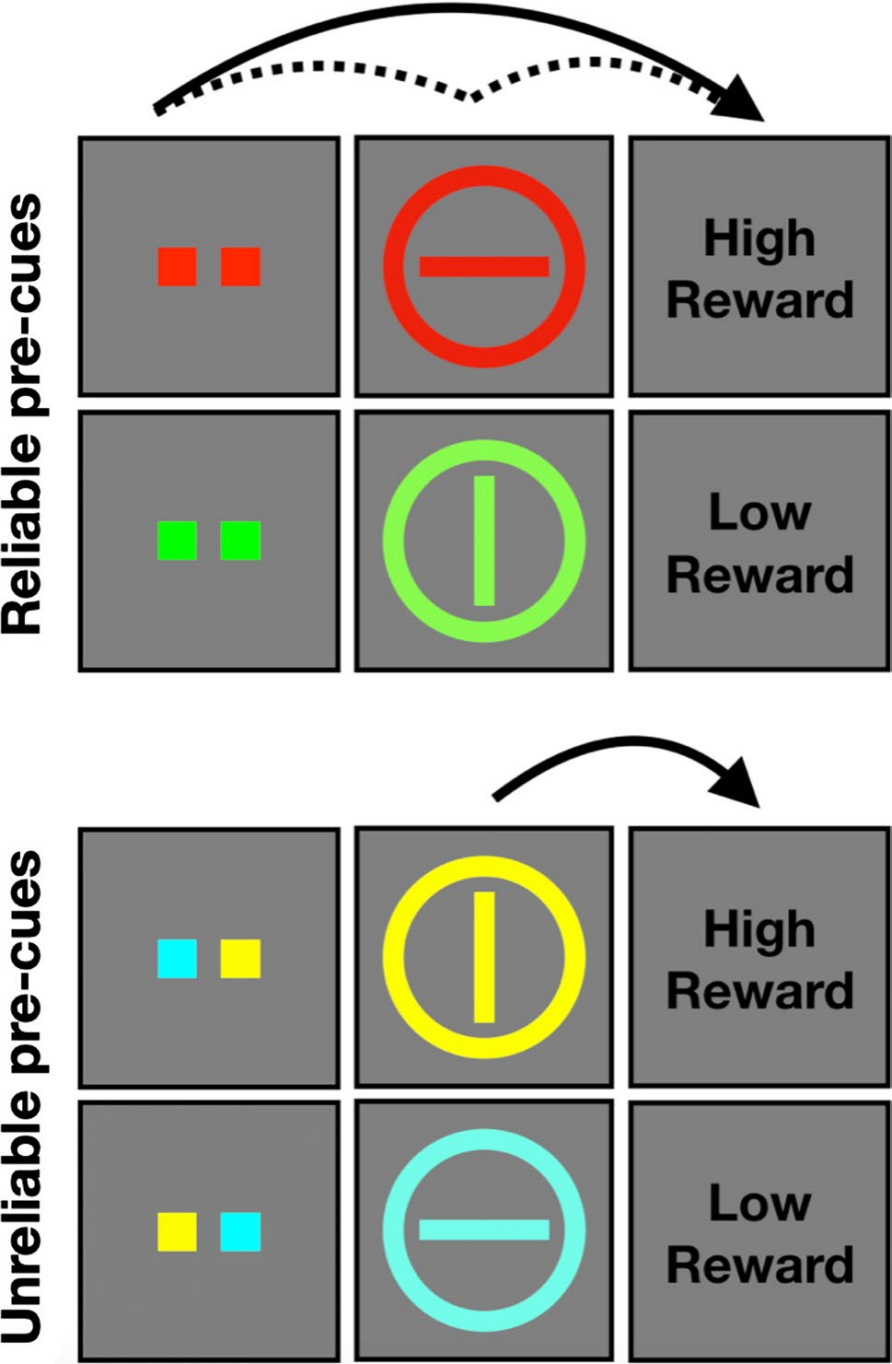
Visual schematic of primary manipulation. In the reliable pre-cue condition (**top panel**), information about reward magnitude is delivered by the pre-cue, and thus, the pre-cue becomes the earliest-reward-predicting stimulus. In the unreliable pre-cue condition (**bottom panel**), information about reward magnitude is delivered by the target itself, and as in the typical VDAC training phase, the training phase target is the earliest-reward-predicting stimulus

Foundational research on dopaminergic reward signaling (e.g., Schultz et al., 1993) has shown a stepwise transfer of the dopamine response from an initially unexpected reward to a cue that precedes and predicts the reward (i.e., the first conditioned stimulus, CS1), and then again from CS1 to a new stimulus (CS2) that precedes and predicts the delivery of CS1. CS2 thus becomes the “earliest reward-predicting” stimulus, evidencing dopamine-mediated higher-order conditioning (Schultz, 2015). Such findings provide a novel behavioral test for the reward-prediction account of VDAC. Our rationale is as follows: given that the color of the training phase target reliably predicts the upcoming reward (e.g., red predicts high and green predicts low reward), if a pre-cue at fixation reliably predicts the color of the subsequent training phase target (e.g., two fixation squares both turn red or both turn green), then the reward prediction signal would be elicited by the pre-cue and not the training phase target itself (i.e., the two colored squares at fixation become the “earliest reward-predicting” stimulus; see Fig. 1, top panel).^1^ When such “reliably pre-cued” targets appear as distractors during the VDAC test phase, they would be unlikely to draw attention and unlikely to interfere with search for the test phase target because these reliably pre-cued distractors were not the earliest reward-predicting stimuli during training.^2^

In the present study, we tested this hypothesis by modifying the traditional VDAC paradigm to include “reliable” pre-cues during the training phase. In addition to the reliable pre-cue condition described above, we also created an “unreliable” pre-cue condition using two additional target colors (e.g., one yellow fixation square and one cyan fixation square; see Fig. 1, bottom panel). These pre-cues signaled an equal probability (0.5) of the upcoming target being yellow or cyan and thus, did not reliably predict the color of the subsequent training phase target. If yellow targets reliably predict high reward and cyan targets reliably predict low reward, for example, then the earliest reward-predicting stimuli (and therefore, the ones that should evoke a unique dopamine-mediated reward prediction), would be the targets themselves and not the pre-cues. When these unreliably pre-cued targets appear as distractors during the test phase, they are expected to capture attention and interfere with search for the test phase target because these unreliably pre-cued distractors were the earliest reward-predicting stimuli during training. This unreliable pre-cue condition closely mimics the parameters of the typical VDAC training phase: at the start of each trial, the target can be one of two known colors, reward can be high or low, and both are uncertain before the appearance of the search array.

Furthermore, if participants use the pre-cues to guide search during the training phase, we should expect to see faster and more accurate search performance when the pre-cue is reliable, relative to unreliable, in line with the extant literature on feature-based attention (Carrasco, 2011).

### Modulating VDAC with higher-order conditioning – expected results

First, test-phase RTs should be slowed when a high-reward-predicting, unreliably pre-cued distractor is present relative to when a low-reward-predicting, unreliably pre-cued distractor is present. We refer to this as *VDAC value-dependency*, in line with the discussion presented in Anderson and Halpern (2017). Second, since neither reliably pre-cued distractor was the “earliest reward-predicting” stimulus during the training phase, neither the high- nor the low-reward distractor is expected to engender capture, resulting in a lack of *VDAC value-dependency* for reliably pre-cued distractors. This pattern would generate an interaction in a 2 × 2 analysis of variance (ANOVA), with pre-cue condition and reward magnitude as within-subjects, repeated-measures factors. Consistent with the expected lack of *VDAC value-dependency* for reliably pre-cued distractors, test-phase RTs are also predicted to be unaffected by the presence of a reliably pre-cued distractor (pooled across reward conditions), relative to the distractor-absent condition (*VDAC elimination*).

### Modulating VDAC with higher-order conditioning – alternative outcomes

A tremendous amount of evidence supports a strict reward-prediction-based explanation of VDAC and undergirds the expected results elucidated above (Anderson et al., 2021). That said, the extant literature on experience-driven attention suggests that *VDAC elimination* may be subject to additional nuance. This prediction is complicated by the fact that reliably pre-cued distractors will have been repeatedly attended in the training phase and thus may still capture attention, in a value-independent manner, due to their histories as sought targets (Anderson et al., 2021; Grubb & Li, 2018; Kim & Anderson, 2019; Sha & Jiang, 2016).

Recent research also suggests that reward magnitude is not always the sole modulator of value-based attention, which complicates the expectation of *VDAC value-dependency* between distractors rendered in unreliable pre-cue colors. When the magnitude of an expected reward (operationalized as expected value, or EV) is equated between different stimuli, uncertainty regarding the trial-to-trial reward outcome can modulate the allocation of attention, with greater uncertainty generating greater capture (Cho & Cho, 2021; Ju & Cho, 2023; Le Pelley et al., 2019). Furthermore, when EV is *not* equated, Le Pelley and colleagues have recently shown that a lower EV cue can induce stronger value-modulated attentional capture if the variance of this lower EV cue sufficiently exceeds that of a higher EV cue (Le Pelley et al., 2023). This intriguing result suggests that when the task environment provides information about different aspects of the reward distribution (both variance and EV), effects of uncertainty can supersede effects of expected value. This finding is particularly relevant for our design because there are two dimensions along which uncertainty is present and information is available: (1) target colors provide information about different magnitudes of reward, with the same amount of uncertainty in all reward conditions; (2) pre-cues provide information about target color, with different amounts of uncertainty between reliable and unreliable pre-cue conditions. If observers were to prioritize target-color uncertainty, which is equivalent for high- and low-reward-predicting targets, we might find equivalent capture by high- and low-reward-predicting, unreliably pre-cued distractors in the test phase (i.e., no *VDAC value-dependency*) that exceeds the amount of capture engendered by reliably pre-cued distractors (i.e., a main effect of pre-cue condition in the 2 × 2 ANOVA described above).

Lastly, the use of eye-tracking in this study provides an additional set of dependent variables (e.g., initial saccades to a distractor, initial saccades to a target), which may provide increased sensitivity to detect overt attentional effects. In the context of reliably measuring *VDAC value-dependency,* it has been suggested that eye-tracking “may have advantages over performance measures such as response time and accuracy” (Anderson & Halpern, 2017, p. 1008). Thus, it is possible to find evidence for some predictions in one domain but not the other.

## Methods

### Overview

Observers completed a modification of the short-training VDAC paradigm developed in Anderson et al., (2011) and used previously in relevant work (e.g., Anderson & Halpern, 2017; Grubb & Li, 2018). Notable changes included: providing pre-cues during the training phase to manipulate target-color uncertainty, and doubling the number of trials in each experimental phase to accommodate the extra conditions. The experiment consisted of 960 total trials, delivered in an approximately 1.5-h session.

### Observers

Eighty-three observers participated in the study. Data from four participants were excluded from all reported analyses for performing at chance accuracy levels in the training phase, test phase, or both, resulting in 79 participants^3^ (age: mean = 19.78, range = 18*–*22 years; gender: 51 F, 26 M, one non-binary, one not reported). Chance accuracy was determined through simulated guessing for 480 trials (the number of trials in each phase of the study): We randomly drew (with replacement) from the set [0,1] 480 times, calculated the mean, repeated this process 10,000 times, and extracted the 95% confidence interval around the mean of the resulting distribution; 0.5438 marked the upper bound, and we used this as our inclusion cutoff.^4^ This study received ethical approval by the Trinity College Institutional Review Board, and informed consent was obtained for each participant.

### Payment

Observers received $10 for participation, as well as a bonus payment that was dependent on accuracy in the training phase (total payment range: $27*–*$39). We rounded up the total payment to the next highest dollar amount (so as to avoid having to use change). The monetary compensation scheme was the same for all participants, but some individuals enrolled in an introductory psychology course were also able to count their participation toward a research participation requirement.

### Training phase

**Fig. 2:**
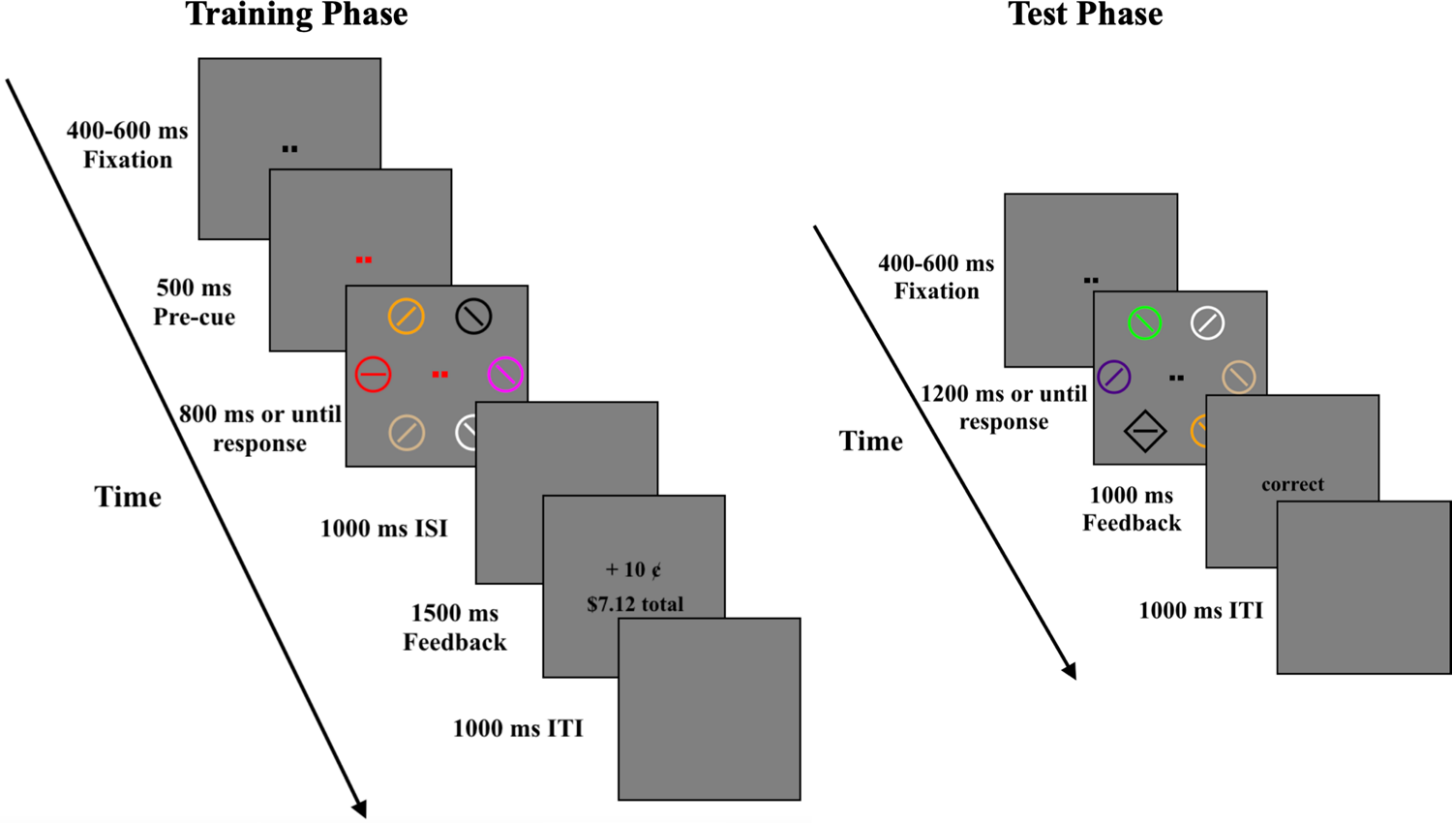
Trial sequences. **Left panel**: Example training phase trial shown with a reliable pre-cue and a red target. **Right panel**: Example test phase trial shown with a diamond target and a green distractor

The task in the training phase was to search for a color-defined target (red, lime, cyan, or yellow^5^) and to report the orientation of the line contained inside (2AFC judgment: vertical or horizontal). Following a randomly selected period of fixation (400, 500, or 600 ms) and a 500-ms pre-cue, a visual search array of six color-defined circles (radii, 1.15 degrees of visual angle (DVA), line thickness = 6 pixels; one target, five distractors located at an eccentricity of 5 DVA) was presented on a gray background. The target was rendered in red, lime, cyan, or yellow, and the distractors’ colors were chosen randomly, without replacement, from black, white, magenta, indigo, orange, and tan. Line segments inside the distractor circles were randomly and independently rotated 45° clockwise or counter-clockwise of vertical (line thickness = 6 pixels). The search array remained on screen until a response was made or until 800 ms, at which point the trial timed out. There was then an inter-stimulus interval of 1,000 ms followed by 1,500 ms of feedback (see *Reward feedback* below for more details)*.* Following each trial was an inter-trial interval of 1,000 ms (see example trial sequence in Fig. 2, left)*.* There were 480 trials in the training phase, delivered as five blocks of 96 trials.

### Pre-cues

Four distinct target colors were used during training (red, lime, cyan, yellow) to accommodate a 2 × 2 within-subjects design that experimentally manipulated pre-cue reliability and reward magnitude. Observers were provided with a cue that was *reliable* (i.e., indicated the exact color of the upcoming target) or *unreliable* (i.e., indicated one of two potential target colors). For each observer, two target colors (e.g., red and lime, as in Fig. 1) were always preceded by reliable cues, and the remaining two target colors (e.g., yellow and cyan, as in Fig. 1) were always preceded by unreliable cues. For reliable cues, both fixation squares were rendered in the color of the subsequent target. For unreliable cues, one fixation square was rendered in one of the unreliable cue colors, and the other fixation square was rendered in the remaining unreliable cue color; the exact spatial location (i.e., left square or right square) was randomly determined on each trial. Within each pair of colors, one color consistently yielded high reward and the other low reward (see *Reward feedback* below). The contingencies between color and reward magnitude, and between color and pre-cue condition, were counterbalanced across observers (i.e., for half the observers, cyan and yellow were reliable pre-cue colors, with cyan predicting high reward for half of this subset of observers; for the other half of observers, red and lime were reliable pre-cue colors, with red predicting high reward for half of this subset of observers). Color-opponent pairs were preserved (i.e., red with lime, cyan with yellow).

#### Reward feedback

Responding correctly to a high-value target within 800 ms yielded a high reward (10¢) with probability 0.8 or a low reward (2¢) with probability 0.2; responding correctly to a low-value target yielded a low reward (2¢) with probability 0.8 or a high reward (10¢) with probability 0.2. If a correct response was made, observers were shown their reward for the current trial, displayed above the total rewards accrued thus far. If the response was made before the deadline but was incorrect, the word “incorrect” was displayed; if no response was made before the deadline, the words “too slow” were displayed.

#### Training phase practice trials

Before beginning the training phase, observers completed 24 practice trials, but no rewards were provided for correct responses; rather, feedback of “correct,” “incorrect,” and “too slow” were displayed for accurate responses, inaccurate responses, and missed responses, respectively.

#### Test phase

The test phase replicated the methodology used in relevant work (e.g., Anderson & Halpern, 2017, Grubb & Li, 2018). Observers were informed that color was irrelevant. The task was to search for a shape-defined target (a diamond among circles or a circle among diamonds, each appearing an equal number of times) and report the orientation of the line contained inside the shape-defined target (vertical or horizontal). The search array remained onscreen until a response was made or until 1,200 ms, at which point the trial timed out. Each element of the array was a unique color.

In half of the trials (value distractor present), one (and only one) of the distractor elements was rendered in a color that was reward-predictive during the training phase (i.e., red, lime, cyan, or yellow, with each appearing an equal number of times). In the other half (value distractor absent), none of the distractor shapes were rendered in a color that was reward-predictive during the training phase. The target (i.e., the shape singleton) was never rendered in a color that was reward-predictive during the training phase; the colors of the target and additional distractors were chosen randomly, without replacement, from black, white, magenta, indigo, orange, and tan. The target and value distractor (when present) were equally likely to appear at all six locations in the stimulus array. Reward feedback was no longer given. Instead, 1,000 ms of visual feedback: “correct,” “incorrect,” and “too slow” was displayed for accurate responses, inaccurate responses, and missed response deadlines, respectively, directly following the response window. Following each trial was an inter-trial interval of 1,000 ms (see trial example, Fig. 2, right). There were 480 trials in the test phase, delivered as five blocks of 96 trials. Before beginning the test phase, observers completed 12 practice trials, with no value distractor present.

#### Dependent variables

Mean RT was calculated for correct trials only, and in line with convention (e.g., Anderson et al., 2011; Anderson & Halpern, 2017), RT distributions were trimmed to remove responses occurring 3 standard deviations above/below the condition mean. Task accuracy (proportion correct; trials in which the deadline was missed were counted as incorrect trials) served as a secondary dependent variable.

**Fig. 3:**
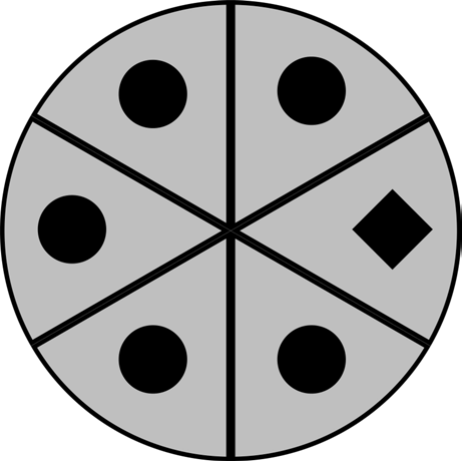
A simplified visualization of the six wedges that contain a task-relevant location at their center

Early work on oculomotor capture used the proportion of first saccades to the target hemifield as the dependent variable (Anderson & Yantis, 2012), while later work has prioritized saccades and dwell time on the distractors themselves (e.g., Anderson & Kim, 2019). Here, we localized the first saccade to one of six pie-shaped “wedges” that emanate from the middle of the screen at 60° angles; the wedges are arranged such that one of the six task locations sits at the center of each wedge (Fig. 3). This allows us to assess whether the first saccade landed in the wedge that contained the target, the wedge that contained one of the pre-cued distractors (if present), or a wedge that contained neither the target nor one of the pre-cued distractors. If no saccade was detected, one cannot determine the location of the *first* saccade. Such trials were coded as NA, which led to their exclusion in the analyses reported below.

#### Eye tracking

Eye movements were recorded using an EyeLink 1000 infrared-video eye tracker (Eyelink, SR Research, Ottawa, Ontario). A 9-point calibration routine was performed before each experimental phase. We used the eyelinkReader package to analyze the EDF files in R Studio (https://github.com/alexander-pastukhov/eyelinkReader).

#### Apparatus

The experiment was programmed in PsychoPy (Peirce & MacAskill, 2018) and run on a 3.0GHz Dual-Core Intel Core i7 Mac Mini; stimuli were displayed on 27.0-in. LED-Lit Dell Gaming Monitor (model: S2716DG). Participants were seated in a darkened experimental testing room and kept their chins in a chin rest 70 cm from the monitor. Responses were collected with a Logitech F310 gaming controller. Participants were instructed to hold the controller with their right hand, to rest their index finger on the top right button, and to rest their thumb on the yellow Y button. The top right button was used to report horizontal judgements, and the yellow Y button was used to report vertical judgements.

##### Bayes factors

We used the BayesFactor package for R to compute the Bayes Factors reported below (https://richarddmorey.github.io/BayesFactor/).

##### Generalized linear mixed-effects (GLME) models

We used the lme4 package for R to compute the GLMEs reported below (https://cran.r-project.org/web/packages/lme4/index.html).

##### Confidence intervals

To compute 95% confidence intervals, we randomly sampled 79 observers (with replacement) and recomputed the mean, within-participant difference for each effect. We then repeated this procedure 10,000 times to build a distribution for each contrast, drawing a new random sample of 79 observers on each iteration. We then extracted the inner 95% of the resultant distribution.

## Results

### Training phase

Reliably pre-cueing target color improved visual search during the training phase. A 2 × 2 ANOVA (with pre-cue condition and reward magnitude as within-subjects, repeated-measures factors) revealed a significant main effect of pre-cue condition for both RT (F(1,78) = 551.2, *p <* .0001, μ_reliable_ = 549.5 ms, μ_unreliable_ = 588.8 ms) and accuracy (F(1,78) = 96.27, *p <* .0001, μ_reliable_ = 88.15%, μ_unreliable_ = 82.07%). That said, there was no evidence for a main effect of reward magnitude (both *p*s >= .894) or pre-cue condition × reward magnitude interaction (both *p*s >= .546). We ran two additional ANOVAs to ensure that there were no effects of reward magnitude that were temporally contingent: (1) the same 2 × 2 ANOVA, but restricted to the last quarter of trials in the training phase, (2) a 2 × 2 × 2 ANOVA with session half as an additional within-subjects, repeated-measures factor. Both ANOVAs confirmed the robustness of the main effect of cue-type for accuracy and RT but did not reveal any main effects of reward magnitude or interactions involving reward magnitude (see Online Supplemental Material for full reporting). In short, pre-cue condition modulated search performance during training, as was expected from the extant literature on feature-based attention (Carrasco, 2011), but the magnitude of the available reward did not.

### Test phase – response time (RT) effects

**Fig. 4:**
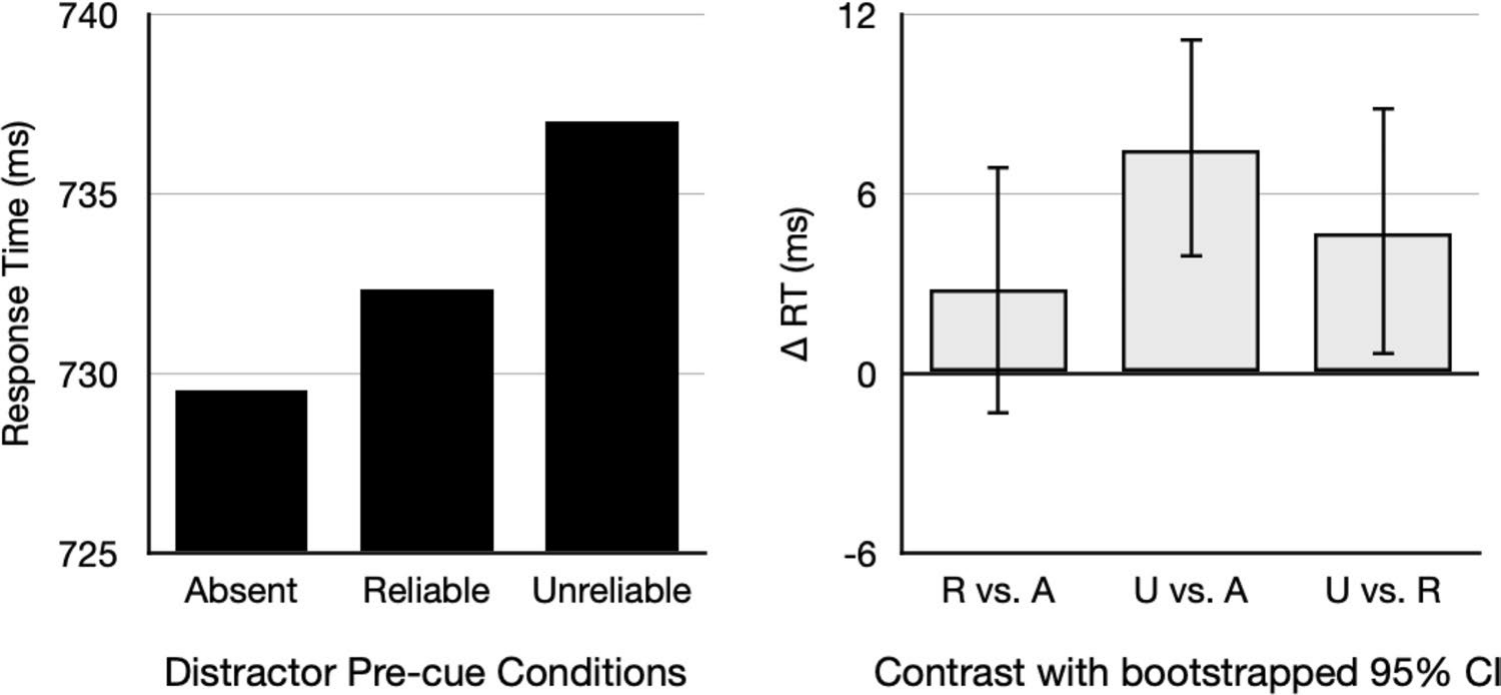
Response time effects. **Left panel**: Condition means (pooled across reward magnitude). **Right panel**: Within-subject contrasts, with bootstrapped 95% confidence intervals. R vs. A: reliably pre-cued distractor vs. distractor-absent. U vs. A: unreliably pre-cued distractor vs. distractor-absent. U vs. R: unreliably pre-cued distractor vs. reliably pre-cued distractor

Here we test the following predictions: (1) *VDAC value dependency* should be observed for unreliably pre-cued distractors, but not for reliably pre-cued distractors, resulting in a pre-cue reliability × reward magnitude interaction. (2) Reliably pre-cued distractors (irrespective of reward magnitude) should not modulate RTs relative to the distractor-absent condition (*VDAC elimination*). To assess the first prediction, we ran a 2 × 2 ANOVA on distractor-present trials (with pre-cue condition and reward magnitude as within-subjects, repeated-measures factors, Table 1). This revealed a significant main effect of pre-cue condition (F(1,78) = 4.954, *p =* .0289, Fig. 4, U vs. R), but no evidence for a main effect of reward magnitude (*p* = .736) or pre-cue condition × reward magnitude interaction (*p *= .226). This main effect of pre-cue condition establishes that unreliably pre-cued distractors slowed RTs relative to reliably pre-cued distractors, but surprisingly, reward magnitude had no impact. This 2 × 2 ANOVA (necessarily) leaves out the distractor-absent condition, so to assess our second prediction and to confirm capture by unreliable distractors (irrespective of reward magnitude), we compared each pre-cue condition with the distractor-absent condition. In line with the prediction of *VDAC elimination*, RTs did not differ between the reliably pre-cued distractor and the distractor-absent conditions (two-tailed paired t-test, t(78) = 1.29, p = .2012, BF_01_ = 3.64, Fig. 4, R vs. A). And as a sanity check, unreliably pre-cued distractors did slow RTs relative to the distractor-absent condition (two-tailed paired t-test, t(78) = 3.94, *p *= .0002, BF_10_ = 119.47, Fig. 4, U vs. A).

**Table 1:** Mean (standard deviation) for all test-phase conditions

Response time (ms)	Distractor-absent	Reliably pre-cued	Unreliably pre-cued
Distractor-absent	729.5 (49.9)		
Low-reward-predicting		731.1 (55.1)	739.4 (51.7)
High-reward-predicting		733.9 (51.5)	735.3 (51.3)
Accuracy (proportion correct)			
Distractor-absent	0.8755 (0.0817)		
Low-reward-predicting		0.8789 (0.0796)	0.8793 (0.0836)
High-reward-predicting		0.8743 (0.0839)	0.8749 (0.0833)

To summarize, we show that unreliably pre-cueing targets during training engendered RT-based capture at test, whereas reliably pre-cueing targets during training eliminated RT-based capture at test. That said, our novel pre-cue manipulation also eliminated *VDAC value dependency* for unreliably pre-cued distractors. Importantly, unreliably pre-cued distractors slowed RTs relative to reliably pre-cued distractors, providing direct, novel evidence for an influence of information reliability on attentional capture in the RT domain.

### Test phase – accuracy effects

A 2 × 2 ANOVA (with pre-cue condition and reward magnitude as within-subjects, repeated-measures factors, Table 1) found no evidence for a main effect of pre-cue condition (*p* = .898), no evidence for a main effect of reward magnitude (*p* = .324), and no evidence for a pre-cue condition × reward magnitude interaction (*p* = .983), alleviating concerns about speed-accuracy trade-offs or attention effects manifesting in the accuracy domain that might complicate the interpretation of the RT-based effects.

### Test phase – oculomotor effects

Participants regularly made saccades while searching for the target in the test phase. We calculated the proportion of trials in which a saccade was detected during the presentation of the search array, and saccades occurred in 75.95% of test phase trials. That said, there was considerable variability across participants: the percentage of trials in which a saccade was detected ranged from 0 to 100, with a median value of 91.25%. It is important to note that a failure to detect a saccade could be due to (1) a saccade genuinely not being made, or (2) eye data not being available. Eye data might not be available on a given trial for at least three reasons: (1) an inability to track at all during the initial calibration (for idiosyncratic reasons like anatomical features of the eye, contacts, glasses, eye-make-up, COVID-era face masks, etc.), (2) movement after initial calibration which resulted in a loss of the pupil, or (3) a temporary loss of the pupil. In this study, we prioritized collection of the RT data. Given that sessions were already 90 min, we only calibrated the eye-tracker at the beginning of the training and test phases (rather than before each block within each phase). We report the remaining oculomotor results with that caveat in place.

Our first set of analyses focus on the distractor-present trials. For each trial, we determined if the first saccade was made to the location of the distractor (see *Dependent variables* in *Methods*) or to a different location (i.e., one of the four remaining non-targets or the target itself). To account for the fact that there was considerable variability across participants with respect to how much eye data each participant could contribute, we fit a generalized linear mixed-effects model to trial-level data:^6^ we modeled the probability of the saccade being made to the location of the distractor with a binomial distribution and a linear model containing a random effect for each participant, a fixed effect for pre-cue condition, a fixed effect for reward magnitude, and a fixed effect for the pre-cue condition × reward magnitude interaction. Consistent with the RT data, the interaction term in this first model was not significant (coefficient = -0.1320, *z* = -1.493, *p* = .1356).

Next, we removed the reward magnitude and cue condition × reward magnitude interaction terms. We modeled the probability of the saccade being made to the location of the distractor with a binomial distribution and a linear model containing a random effect for each participant and a single fixed effect for pre-cue condition (1 if the distractor was unreliably pre-cued, 0 if the distractor was reliably pre-cued). Consistent with the RT data, we found that the probability of saccading to the distractor location increased when the distractor had been unreliably pre-cued during training (coefficient = 0.1053, *z* = 2.381, *p* = .0172). For completeness, we also fit a model that contained a random effect for each participant and a single fixed effect for reward magnitude. As expected, we found no impact of reward magnitude on the probability of saccading to the distractor location (coefficient = 0.0390, *z* = 0.883, *p* = .377).

These results establish that pre-cue condition, but not reward magnitude, modulated the probability with which a distractor drew initial eye movements. This converges with the findings in the RT domain, but do we find evidence of *VDAC elimination* in the oculomotor domain? To answer this question, we determined if the first saccade was made to the target location, allowing us to include the distractor-absent trials. We then modeled the probability of a saccade being made to the target location with a binomial distribution and a linear model that contained a random effect for each participant and a single fixed effect for distractor presence (1 if the reliably pre-cued distractor was present on that trial, 0 if not; unreliably pre-cued distractor-present trials were excluded for this analysis). Inconsistent with the RT data, however, we found that the probability of saccading to the target significantly decreased when the reliably pre-cued distractor was present (coefficient = -0.1039, *z* = -3.385, *p* = .0007). For completeness, we fit the same model to the unreliably pre-cued distractor-present trials (this time excluding the reliably pre-cued distractor-present trials). As expected and consistent with the RT data, the probability of saccading to the target significantly decreased when the unreliably pre-cued distractor was present (coefficient = -0.1127, *z* = -3.692, *p* = .0002).

One strength of the trial-level generalized linear mixed effects (GLME) modeling approach is that it accounts for the fact that some participants have substantially less eye data. That said, some readers might object that this approach overweights the contributions of the participants who had more eye data, which could bias the results if these participants differ in some systematic way. To address this concern, we calculated the proportion of trials in which the first saccade was made to the location of the distractor (as opposed to some other location), separately for each pre-cue condition and separately for each participant. For the distractor-absent trials, we calculated the proportion of trials in which the first saccade was made to any of the non-target locations and divided this value by 5 to establish a baseline rate of having the first saccade land at any one of the non-target locations. Below, we compare condition-based differences in these proportions under two scenarios: (1) requiring that there be saccades detected on at least 10% of the trials in each condition (74 participants met this criteria), (2) requiring that there be saccades detected on at least 50% of the trials in each condition (63 participants met this criteria). We use the 10% saccade detection scenario to allow for the inclusion of as many participants as possible; we use the 50% saccade detection criteria to alleviate concerns that overly noisy subject-level estimates in the 10% detection case could bias the statistical results. We concede that these are arbitrary cut-offs, and we note that our goal is simply to present the evidence in multiple ways.

Using this approach, we observed a pattern of attentional allocation that was consistent with the GLME results. First, we again found that unreliably pre-cued distractors attracted more overt attention than reliably pre-cued distractors (paired t-tests, 10% criteria: t(73) = 2.42, *p* = .0181; 50% criteria: t(62) = 2.18, *p* = .0332). Second, we found that both reliable and unreliable distractors drew initial fixations above the baseline rates established by the distractor-absent trials (paired t-tests, reliable, 10% criteria: t(73) = 7.21, *p* < .0001; 50% criteria: t(62) = 7.15, *p* < .0001; unreliable, 10% criteria: t(73) = 9.98, *p* < .0001; 50% criteria: t(62) = 9.58, *p* < .0001).

Finally, we asked: Is there a relationship between the amount of oculomotor capture and the amount of RT-based capture observed across individual observers? In short, the magnitude of oculomotor capture (OC) was significantly correlated with the magnitude of the RT-based capture (RTC) but only for unreliably pre-cued distractors (unreliable minus absent OC ~ unreliable minus absent RTC, 10% criteria: r(72) = 0.267, *p* = .0215; 50% criteria: r(61) = 0.282, *p* = .0251; *p*s for both criteria assessing reliably pre-cued distractors > .6938).

Eye-tracking data provided results that were both consistent with and inconsistent with the RT-based findings. In terms of consistent findings, unreliably pre-cued distractors attracted more overt attention than reliably pre-cued distractors, providing another piece of direct evidence for the influence of information reliability on attentional capture. Additionally, an individual differences analysis linked changes in oculomotor capture to changes in RT-based capture, but only when the distractor had been unreliably pre-cued. In terms of inconsistent findings, our second set of GLME analyses revealed that initial fixations on the target were reduced by the presence of both kinds of distractors, suggesting that attentional capture was not *entirely* eliminated by pre-cueing the target during training. Failure to find *VDAC elimination* in the oculomotor domain was corroborated in the model-free analysis, which showed that the proportion of first saccades to reliable distractors exceeded the baseline rate established by the distractor-absent trials.

## Discussion

In this study, we modified Anderson and colleagues’ short-training VDAC paradigm (Anderson et al., 2011) to include pre-cues that signaled reliable or unreliable information about the trial-to-trial color of the training phase search target. Reliable pre-cues indicated the upcoming target color with certainty, whereas unreliable pre-cues indicated the target was equally likely to be one of two distinct colors, thus providing uncertain information about the upcoming target color. As in the traditional VDAC paradigm, the color of the training phase target was predictive of reward magnitude. In our modification, one of the reliable pre-cue colors and one of the unreliable pre-cue colors were predictive of high reward, while the other two colors in each pre-cue condition were predictive of low reward. We then tested for VDAC in a traditional test phase.

We found that manipulating the reliability of target-color information during training modulated the allocation of value-driven attention in three distinct ways. First, in both the RT and oculomotor domains our training phase manipulation eliminated *VDAC value-dependency* between distractors rendered in colors that had been unreliably pre-cued during training but preserved their ability to draw attention in a reward-magnitude independent manner*.* Second, the presence of distractors rendered in unreliably pre-cued colors (pooled across reward magnitude conditions) slowed RTs and increased the probability of a saccade to the distractor location relative to distractors rendered in reliably pre-cued colors, evidencing a direct impact of information reliability on attentional capture, in both the RT and oculomotor domains. Third, the presence of distractors rendered in colors that had been reliably pre-cued during training had no impact on search time in the test phase (*VDAC elimination* in the RT domain).^7^ Taken together, the three results suggest that target-color uncertainty, rather than reward magnitude, played a critical role.

Before we discuss the effect of information reliability on attentional capture in more detail, we would like to briefly address the fact that the presence of reliably pre-cued distractors did not slow search for the test phase target. These distractors had extensive histories as sought targets (Anderson et al., 2021), and one might reasonably argue that their histories were further enriched by the benefit of feature-based attention, which facilitated both faster and more accurate search performance in the training phase. That they did not slow search in the test phase is particularly striking, especially given previous work from our lab and others (e.g., Grubb & Li, 2018; Kim & Anderson, 2019; Sha & Jiang, 2016) that has documented the robustness of RT-based capture resulting from a “history as a sought target.” Our results in this study suggest that a history of target-seeking, in and of itself, is not always sufficient to engender the reflexive allocation of experience-driven attention as measured by changes in RT.

That said, the presence of reliably pre-cued distractors did impact the allocation of overt attention. First, the proportion of initial saccades made to a reliably pre-cued distractor exceeded the baseline estimates derived from the distractor-absent trials. Second, the probability of saccading to the target decreased when a reliably pre-cued distractor was present. Thus, it would be inaccurate to say that reliably pre-cueing the target during training completely eliminated attentional capture. A history of target seeking did impact the allocation of attention in this study, but it was only detectable using eye-tracking. It is important to note, however, that reliably and unreliably pre-cued distractors were “sought as targets” an equal number of times in the training phase. Thus, we can be confident that the RT and oculomotor differences *between them* are due to the target-color uncertainty manipulation and not their histories as sought targets.

Given the failure to find *VDAC value-dependency* for distractors rendered in unreliably pre-cued colors, our results (taken at face value) are inconsistent with a straightforward reward-prediction account of VDAC. One simple explanation is that our training phase, with four different colors each signaling two different kinds of reward in a probabilistic manner, is too complex and rendered observers insensitive to differences in reward magnitude. This would be consistent with a study by Marchner and Preuschhof (2018), who introduced a clever manipulation to the literature to assess the controversy around search history alluded to above. In their paradigm, three target color conditions were used: the traditional probabilistically determined high- and low-reward target colors and an additional unrewarded target color. Interestingly, they found no difference in capture between high- and low-reward-predicting distractors, though they did find a difference in capture between high- and no-reward-predicting distractors (convincingly ruling out a history-as-sought-target interpretation). They additionally found capture between low- and no-reward-predicting distractors during the first block of the test phase. One speculation Marchner and Preuschhof noted was that the higher working memory load required to create color-reward associations for three conditions, rather than two, could underlie the lack of value-dependency. That we found equivalent capture by unreliably pre-cued distractors, irrespective of reward magnitude, is consistent with the results of Marchner and Preuschhof, and we concede that higher working memory demands during a more complex training phase may provide a plausible explanation for the failure to find *VDAC value-dependency*.

A different interpretation of the Marchner and Preuschhof results concerns the uncertainty of the trial-to-trial reward outcomes. In their study, the high-reward and low-reward training phase targets had different expected values (EV), but they had identical variances (i.e., the extent of deviation from the mean value) and identical entropy (i.e., amount of information provided by an outcome). Since the no-reward target always predicted no reward, it had an EV of zero and no associated uncertainty. Thus, the three targets differed along two reward-relevant dimensions: (1) they each had a different EV, and (2) the variance and entropy of the high- and low-reward targets were equated and exceeded that of the no-reward target. As presented in the *Introduction*, recent work from Le Pelley and colleagues and Cho and colleagues has demonstrated the modulatory power that reward uncertainty can have on value-based attention (Cho & Cho, 2021; Ju & Cho, 2023; Le Pelley et al., 2019). If the uncertainty of the reward distribution was implicitly prioritized over the EV dimension (e.g., Le Pelley et al., 2023), then one would expect the exact results reported in Marchner and Preuschhof (2018): statistically indistinguishable capture from the two uncertainty-associated distractors and no capture from the certainty-associated distractor.

In our design, there was a salient difference in uncertainty during the training phase that aligns well with the pattern of results we observed in the test phase, but it concerns informational uncertainty pertaining to target color rather than the EV, variance, or entropy of the trial-to-trial reward outcomes (which were matched during the training phase for reliably and unreliably pre-cued targets). The training phase results confirmed that participants voluntarily used, and were thus sensitive to, the information signaled by the pre-cues. This is in contrast to the EVs of the training phase targets, which did not modulate training phase performance.

Why might target-color uncertainty have been a salient dimension in our study? A nascent literature on the neuroscience of information-seeking (Charpentier & Cogliati Dezza, 2022) provides one potential framework in which to interpret our results. In their review, Charpentier and Cogliati Dezza make the case that “information is valuable, similar to primary or monetary rewards”, which “is reflected in the brain via a common neural code for reward and information.” One motive for information-seeking that the authors address is instrumentality, which they define as “[t]he potential of information to improve future decisions, actions, and outcomes.” To obtain monetary reward in our training phase, participants must use color to localize and discriminate the line inside in the target, and thus, information about target color provides instrumental value in this context. In the reliable pre-cue condition, such information is provided by the pre-cue in advance of the search array. But in the unreliable pre-cue condition, observers must wait for the appearance of the search array, and the instrumental value of target-color information is provided by the search target itself. Thus, unreliably pre-cued targets consistently provided instrumental value in the training phase, and they did so in a manner that was independent of the reward magnitude signaled by a specific color. This is in contrast to reliably pre-cued targets whose instrumental value was provided by the pre-cues. We note that the context in which each set of training phase colors provided instrumental value was quite different (i.e., a gray screen with two colored squares presented at fixation vs. the multi-color, multi-object training phase search array). Importantly, the visual context of the test phase is similar to the training phase search array but quite distinct from the pre-cue presentation screen, which is likely to matter given that VDAC has been shown to be context-specific (Anderson, 2015). To put this back in the language that is typical of VDAC studies, our results show that the presence of stimuli that provided instrumental value during the training phase slowed response times and drew more initial saccades during the test phase, relative to stimuli that provided no such instrumental value during training.

One could reasonably argue that the color of the training phase target provides instrumental value in all VDAC studies, so we want to make clear that our claim is not that target color information always plays such a role. Instead, it seems plausible that manipulating the relative reliability of target color information in our training phase signaled that target color was a feature dimension about which information could be gained. That is, pre-cueing targets with a cue that consistently resolved uncertainty in some cases (i.e., reliable pre-cues) but not others (i.e., unreliable pre-cues) may have made the instrumental value of target color more salient than is typically the case. Information-seeking as an explanatory framework has featured prominently in recent literature, establishing that uncertainty impacts VDAC and other forms of value-modulated attention (e.g., Cho & Cho, 2021; Ju & Cho, 2023; Le Pelley et al., 2019). Our results are consistent with this body of work.

To the best of our knowledge, no previous study has manipulated the reliability of target-color information in this manner, and the results from this study have contributed to our understanding of value-driven attention in multiple ways. We showed that we can eliminate *VDAC value-dependency* with the right experimental manipulation, that we can modulate the amount of RT-based and oculomotor capture engendered by two pairs of former targets despite their reward distributions being matched on EV, variance, and entropy, and that “history as a sought target” is not always sufficient to engender the reflexive allocation of experience-driven attention in the RT domain. Many important questions remain, but an emerging literature on the neuroscience of information-seeking provides an exciting avenue for future research.

### Supplementary Information

Below is the link to the electronic supplementary material.

Supplementary file1 (DOCX 23 KB)

## Data Availability

Upon publication, data, analysis scripts, and a series of video tutorials explaining the R Studio code will be available for download on the corresponding author’s website (www.attentionPerceptionDecision.com/MCLG_2024).

## Footnotes

1 We thank an anonymous reviewer of a previous Registered Report attempt for clearly articulating this prediction.

2 Note that previous work on experience-dependent attentional capture (e.g., Grubb & Li, 2018) complicates this prediction. Furthermore, recent work on the role of reward uncertainty has complicated existing theory even more. The full story cannot be one of simple reward-magnitude based prediction and prediction error because one can match stimuli on reward magnitude, manipulate the uncertainty of the reward distribution (variance, entropy, etc.), and show that greater uncertainty leads to greater attentional capture (Cho & Cho, 2021; Ju & Cho, 2023; Le Pelley et al., 2019). These concerns are discussed in more detail in the subsection *Modulating VDAC with higher-order conditioning – alternative outcomes.*

3 A stopping rule and an accompanying data analysis plan were pre-registered on the Open Science Framework (https://doi.org/10.17605/OSF.IO/B4D2U.) After 70 observers met the inclusion criteria, we satisfied all of the criteria for outcome #3 in the Sample Size section of the pre-registration. Given the uncertainty of when we would reach the stopping rule, we had six participants who were already scheduled and were in the process of scheduling times for an additional three participants. We committed to running these nine additional participants (before seeing any of their data), which led to a total sample size of 79 observers. Importantly, however, this preregistration was written with a different set of explanatory hypotheses in mind (a focus on feature-based attention and the impact of history as a former search target). Reviewer feedback from a Registered Report attempt, a more thorough review of the evidence in support of a reward-prediction error account of VDAC in the literature, and the outcomes of the current study have alleviated our former concerns about the potential impact of target history in the interpretation of VDAC studies. However, given this shift in framing, we no longer feel justified in reporting the pre-registered analyses as such, and we follow a different analysis approach in this manuscript in line with reviewer feedback.

4 With infinite data, this simulated guessing procedure will converge on 0.5.

5
Color names follow the web/X11 standards, see https://www.w3schools.com/Colors/colors_names.asp

6 This ensures that participants who have very little data don’t exert an outsized influence on the fixed effects estimates.

7 That distractors rendered in colors that had been reliably pre-cued during training did engender oculomotor capture is addressed in the following paragraph.

## References

[CR1] Anderson BA (2015). Value-driven attentional priority is context specific. Psychonomic Bulletin & Review.

[CR2] Anderson BA (2018). Neurobiology of value-driven attention. Current Opinion in Psychology.

[CR3] Anderson BA, Halpern M (2017). On the value-dependence of value-driven attentional capture. Attention, Perception, & Psychophysics.

[CR4] Anderson BA, Kim H (2019). On the relationship between value-driven and stimulus-driven attentional capture. Attention, Perception, & Psychophysics.

[CR5] Anderson BA, Yantis S (2012). Value-driven attentional and oculomotor capture during goal-directed, unconstrained viewing. Attention, Perception, & Psychophysics.

[CR6] Anderson BA, Laurent PA, Yantis S (2011). Value-driven attentional capture. Proceedings of the National Academy of Sciences.

[CR7] Anderson BA, Laurent PA, Yantis S (2014). Value-driven attentional priority signals in human basal ganglia and visual cortex. Brain Research.

[CR8] Anderson BA, Kim H, Kim AJ, Liao MR, Mrkonja L, Clement A, Gregoire L (2021). The past, present, and future of selection history. Neuroscience & Biobehavioral Reviews.

[CR9] Anderson, B. A., Kuwabara, H., Wong, D. F., Gean, E. G., Rahmim, A., Brasic, J. R., ..., Yantis, S. (2016). The Role of Dopamine in Value-Based Attentional Orienting. *Curr Biol, 26*(4), 550-555. 10.1016/j.cub.2015.12.062 10.1016/j.cub.2015.12.062 PMC4767677 26877079

[CR10] Awh E, Belopolsky AV, Theeuwes J (2012). Top-down versus bottom-up attentional control: a failed theoretical dichotomy. Trends in Cognitive Sciences.

[CR11] Carrasco M (2011). Visual attention: the past 25 years. Vision Research.

[CR12] Charpentier C, CogliatiDezza I, Dezza IC, Schulz E, Wu C (2022). Information-Seeking in the Brain. The Drive for Knowledge: The Science of Human Information Seeking.

[CR13] Cho SA, Cho YS (2021). Uncertainty modulates value-driven attentional capture. Attention, Perception, & Psychophysics.

[CR14] Daw N, Tobler P, Glimcher PW, Fehr E (2013). Value Learning through Reinforcement: The Basics of Dopamine and Reinforcement Learning. Neuroeconomics: Decision making and the brain.

[CR15] Failing M, Theeuwes J (2018). Selection history: How reward modulates selectivity of visual attention. Psychonomic Bulletin & Review.

[CR16] Grubb MA, Li Y (2018). Assessing the role of accuracy-based feedback in value-driven attentional capture. Attention, Perception, & Psychophysics.

[CR17] Ju J, Cho YS (2023). The modulation of value-driven attentional capture by exploration for reward information. Journal of Experimental Psychology: Learning, Memory, and Cognition.

[CR18] Kim H, Anderson BA (2019). Dissociable neural mechanisms underlie value-driven and selection-driven attentional capture. Brain Research.

[CR19] Le Pelley ME, Pearson D, Porter A, Yee H, Luque D (2019). Oculomotor capture is influenced by expected reward value but (maybe) not predictiveness. Journal of Experimental Psychology (Hove).

[CR20] Le Pelley M, Pearson D, Chong A (2023). Reward variance outweighs reward value in modulating capture of visual attention. Journal of Vision.

[CR21] Marchner JR, Preuschhof C (2018). Reward history but not search history explains value-driven attentional capture. Attention, Perception, & Psychophysics.

[CR22] Peirce J, MacAskill M (2018). Building experiments in PsychoPy.

[CR23] Sali AW, Anderson BA, Yantis S (2014). The role of reward prediction in the control of attention. Journal of Experimental Psychology: Human Perception and Performance.

[CR24] Schultz W (2015). Neuronal reward and decision signals: from theories to data. Physiological reviews.

[CR25] Schultz W, Apicella P, Ljungberg T (1993). Responses of monkey dopamine neurons to reward and conditioned stimuli during successive steps of learning a delayed response task. Journal of Neuroscience.

[CR26] Schultz W, Dayan P, Montague PR (1997). A neural substrate of prediction and reward. Science.

[CR27] Sha LZ, Jiang YV (2016). Components of reward-driven attentional capture. Attention, Perception, & Psychophysics.

