# Supplementary material for: Manipulating the reliability of target-color information modulates value-driven attentional capture
Source: Atten Percept Psychophys. 2024 Mar 27;86(4):1108–19. doi: 10.3758/s13414-024-02878-7 (PMC11093855; doi:10.3758/s13414-024-02878-7)
Supplement: Supplementary file 1 — Supplementary file1 (DOCX 23 KB) [file 13414_2024_2878_MOESM1_ESM.docx]

# Supplemental Material Training Phase RT Data

|  | **F** | ***p*** |
| --- | --- | --- |
| **Pre-Cue** | 273.6 | < .0001 |
| **Reward Magnitude** | 0.021 | 0.884 |
| **pre-cue X reward magnitude** | 0.532 | 0.468 |

**Table S1.** 2x2 ANOVA on training phase RT data (last quarter) with pre-cue and reward magnitude as within-subjects, repeated-measures factors.

|  | **F** | ***p*** |
| --- | --- | --- |
| **Pre-Cue** | 512.6 | < .0001 |
| **Reward Magnitude** | .0395 | .843 |
| **Session Half** | 51.84 | < .0001 |
| **pre-cue X reward magnitude** | .5793 | .449 |
| **pre-cue X session half** | 2.406 | .125 |
| **reward magnitude X session half** | .1269 | .723 |
| **pre-cue X reward magnitude**  **X session half** | .2518 | .617 |

**Table S2.** 2x2x2 ANOVA on training phase RT data with pre-cue, reward magnitude, and session half as within-subjects, repeated-measures factors.

# Training Phase Accuracy Data

|  | **F** | ***p*** |
| --- | --- | --- |
| **Pre-Cue** | 17.9 | < .0001 |
| **Reward Magnitude** | 0.995 | 0.322 |
| **pre-cue X reward magnitude** | 0.314 | 0.577 |

**Table S3.** 2x2 ANOVA on training phase accuracy data (last quarter) with pre-cue and reward magnitude as within-subjects, repeated-measures factors.

|  | **F** | ***p*** |
| --- | --- | --- |
| **Pre-Cue** | 96.27 | < .0001 |
| **Reward Magnitude** | .0037 | 0.952 |
| **Session Half** | 93.96 | < .0001 |
| **pre-cue X reward magnitude** | .081 | .777 |
| **pre-cue X session half** | 19.55 | < .0001 |
| **reward magnitude X session half** | 1.76 | .189 |
| **pre-cue X reward magnitude**  **X session half** | .0378 | .846 |

**Table S4.** 2x2x2 ANOVA on training phase accuracy data with pre-cue, reward magnitude, and session half as within-subjects, repeated-measures factors.
